# Triptolide Induces Growth Inhibition and Apoptosis of Human Laryngocarcinoma Cells by Enhancing p53 Activities and Suppressing E6-Mediated p53 Degradation

**DOI:** 10.1371/journal.pone.0080784

**Published:** 2013-11-14

**Authors:** Fei Zhao, Weiwei Huang, Tamgue Ousman, Bin Zhang, Yangyang Han, Daguia Zambe John Clotaire, Chen Wang, Huanhuan Chang, Huanan Luo, Xiaoyong Ren, Ming Lei

**Affiliations:** 1 Key Laboratory of Agricultural Molecular Biology, College of Life Sciences, Northwest A&F University, Yangling, Shaanxi Province, People's Republic of China; 2 Department of Otolaryngology-Head and Neck Surgery, the Second Hospital, Xi’an Jiao Tong University, Xi’an, Shaanxi Province, People's Republic of China; German Cancer Research Center, Germany

## Abstract

Triptolide, an active compound extracted from Chinese herb Leigongteng (*Tripterygium wilfordii* Hook* F*.), shows a broad-spectrum of anticancer activity through its cytotoxicity. However, the efficacy of triptolide on laryngocarcinoma rarely been evaluated, and the mechanism by which triptolide-induced cellular apoptosis is still not well understood. In this study, we found that triptolide significantly inhibited the laryngocarcinoma HEp-2 cells proliferation, migration and survivability. Triptolide induces HEp-2 cell cycle arrest at the G1 phase and apoptosis through intrinsic and extrinsic pathways since both caspase-8 and -9 are activated. Moreover, triptolide enhances p53 expression by increasing its stability via down-regulation of E6 and E6AP. Increased p53 transactivates down-stream target genes to initiate apoptosis. In addition, we found that short time treatment with triptolide induced DNA damage, which was consistent with the increase in p53. Furthermore, the cytotoxicity of triptolide is decreased by p53 knockdown or use of caspases inhibitor. In conclusion, our results demonstrated that triptolide inhibits cell proliferation and induces apoptosis in laryngocarcinoma cells by enhancing p53 expression and activating p53 functions through induction of DNA damage and suppression of E6 mediated p53 degradation. These studies indicate that triptolide is a potential anti-laryngocarcinoma drug.

## Introduction

Cancer is one of the main causes of human death worldwide, and its incidence increases year by year [1]. Researchers have made great efforts to fight against cancer, but the outcomes still cannot ameliorate its burden. Among numerous cancer types, those most frequently diagnosed and with the greatest casualties, such as lung cancer and breast cancer, receive the greatest research attention. Consequently, cancer types with low incidence and mortality, like laryngocarcinoma, are neglected. Laryngocarcinoma is mostly squamous cell carcinomas that is primarily formed or transferred in larynx, and usually associates with pharynx cancer. It’s estimated that there are 12,260 new cases of laryngocarcinoma in America in 2013 including 3,630 fatal cases [2]. Although the incidence and mortality of laryngocarcinoma are low, being 3.4 and 1.1 per 100,000 people each year, respectively [2], its associated complications, such like trachyphonia, dyspnea, cough and dysphagia, cause lots of pain to the patients, whereby require effective therapeutic treatment. The underlying cause of laryngocarcinoma is still not determined. In addition to smoking, drinking and air pollution, high-risk human papillomavirus (HPV) infection is also considered as a major etiologic factor for the occurrence of laryngocarcinoma. HPV encodes oncoproteins E6 and E7, which target tumor suppressor p53 and pRb respectively and induce carcinogenesis [3–6]. 

The transcriptional factor p53, one of the most important tumor suppressor in cells, protects normal cell growth and initiates malignant cell death [7]. p53 can be activated by a variety of cytotoxic stresses, such as DNA damage induced by ionic irradiation and chemicals, activation or mutation of oncogenes, hypoxia and virus infection [8]. In unstressed cells, p53 level and activity is strictly controlled especially by the ubiquitin E3 ligase MDM2, which binds p53 directly and continuously mediates p53 ubiquitination and proteasomal degradation [8]. When cells suffer toxic stresses, p53 is activated. Through both transcription-dependent and -independent mechanisms, p53 induces cell growth arrest, DNA repair, senescence and apoptosis [8,9]. p53 is found to be mutated in about 50% of human cancers underscoring its importance in tumor suppression. These mutations result in p53 losing its activity or even functioning as an oncoprotein [10]. In HPV positive cells, HPV oncoprotein E6 represses p53 activation by directly binding p53 and inducing its ubiquitination and degradation through E6AP, an E6 associated protein and an ubiquitin ligase [4,5]. Due to its anti-tumor activity, p53 or its regulators are appealing targets for anti-cancer drug development [11]. Several compounds targeting p53 have been found with potent anti-tumor activity, such as reactivation of p53 and induction of tumor cells apoptosis (RITA) and Nutlins. RITA binds p53 N-terminal domain with high affinity and induces many cancer cells apoptosis in a p53-dependent manner *in vitro* and in vivo [12]. Nutlins effectively disturbs the p53-MDM2 interaction by binding MDM2 whereby induce and activate p53, and show anti-tumor activity [13]. These studies indicate that targeting p53 is an effective strategy to fight against cancers. 

Triptolide is a diterpene lactone extracted from Chinese medicinal herb leigongteng (*Tripterygium wilfordii* Hook* F.*) which has been used to treat inflammatory and immunological diseases for centuries, such as rheumatoid arthritis (RA) [14]. As the major active component in leigongteng, triptolide has attracted high attention for its multiple activities, especially for its anti-tumor effect [14]. Triptolide has shown a broad-spectrum of anti-tumor effect and cytotoxicity on almost all kinds of cancers, including lung cancer [15], pancreatic cancer [16], breast cancer [17], ovary cancer [18], renal cancer [19], glioblastoma [20], thyroid cancer [21], leukemia [22], colon cancer [23]. Our previous study also found that triptolide shows effective anti-PCa activity [24]. Triptolide inhibits cancer cells and xenografted tumors growth in vitro and in vivo, induces apoptosis [25], and suppresses angiogenesis [26] and tumor metastasis [27]. Triptolide shows high potential of anti-tumor effect with IC_50_ at nanomole level on many cancer cells [28].These researches indicate triptolide may be a potential anti-cancer drug. 

The anti-tumor effect of triptolide is mainly dependent on its cytotoxicity. Triptolide induces apoptosis on many cancer cells with all features of apoptosis, such as DNA fragmentation, cytochrome C release, caspases activation, PARP cleavage, and apoptotic proteins expression change [14]. A number of studies demonstrate that triptolide down-regulates many genes expression, including transcriptional factors and so on [14]. This effect is believed to result from the direct interaction of triptolide with XPB, a component of the transcriptional complex TFII H, leading to the inhibition of the global transcription [28]. However, the precise mechanism of apoptosis initiation by triptolide is still undetermined. 

In this study, we examined the effect of triptolide on human laryngocarcinoma cells and investigated the underlying mechanism of apoptosis induction by triptolide. Our results demonstrated triptolide may induce DNA damage and triggers the accumulation of p53 partially through the enhancement of protein stability by inhibiting the E6/E6AP mediated p53 proteasomal degradation in HEp-2 cells. This leads to intrinsic and extrinsic apoptotic pathway activation and results in laryngocarcinoma cells growth suppression and apoptosis. Overall, our findings suggest that triptolide is a potential anti- laryngocarcinoma drug, which induces cells growth inhibition and apoptosis through activating p53 function and suppressing HPV oncoprotein E6 activity. 

## Results

### Triptolide inhibits the growth, migration and survivability of laryngocarcinoma cells

**Figure 1:**
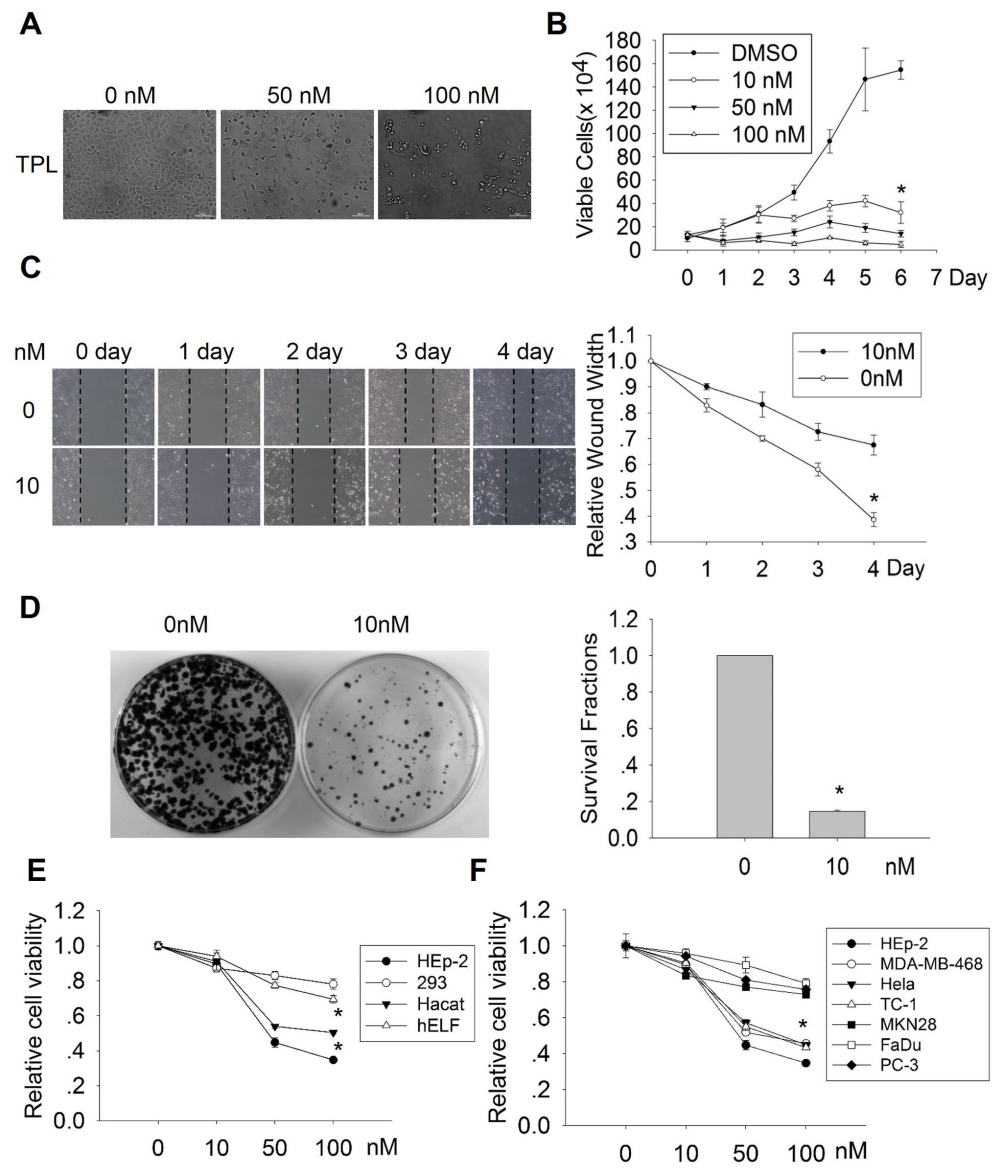
Effects of triptolide on the growth, migration and survivability of laryngocarcinoma cells. (A) Morphology change of triptolide treated HEp-2 cell. HEp-2 cells were treated with various does of triptolide for 24h and cell images were captured with an invert microscope. (B) Effect of triptolide on the growth of HEp-2 cell. HEp-2 cells were treated with indicated doses of triptolide, viable cells were counted every 24h for 7 days. (C) Effect of triptolide on the migration of HEp-2 cell. Scratches were made by using a 200ul pipette tip and treated with various does of triptolide, and imaged every 24h. The edges of the scratches on the pictures were marked with dashed lines. The wound width was measured and the relative wound width was presented. (D) Effect of triptolide on the survivability of HEp-2 cell. 1000 cells were seeded in 60mm dishes and cultured in medium with 10nM triptolide for 2 to 3 weeks. After fixed and stained, pictures of cell colonies were captured with ChemiDoc XRS+ imaging system. The numbers of colonies were counted and the survival fractions were calculated. (E) Effect of triptolide on HEp-2, 293, Hacat and hELF cells. (F) Effect of triptolide on HEp-2, MDA-MB-468, Hela, TC-1, MKN28, FaDu and PC-3 cells. Cells were treated with indicated doses of Triptolide and cells viability was determined by CCK8 assay. The asterisks indicate P <0.05.

To investigate the activity of triptolide on laryngocarcinoma cells proliferation, HEp-2 cells were treated with indicated doses of triptolide and subjected to various assays. As shown in Figure 1, after treatment with triptolide, the morphology of HEp-2 cells shrunk to spindle or even round shape from polygon, and cell density decreased markedly following treatment with increasing doses of triptolide (Figure 1A), which suggests that triptolide could inhibit HEp-2 cells growth and even induce cell death. To exactly analyze the effect of triptolide on laryngocarcinoma cells, we performed growth curve assay. The result showed that triptolide inhibits the proliferation of HEp-2 cells in a dose-dependent manner, and shows significant cell growth inhibition even at 10 nM (Figure 1B). We further carried out cell viability assay to qualify the efficacy of triptolide on HEp-2 cells proliferation. The result showed that the IC50 value of triptolide is 39.5 nM. We also detected the effect of triptolide on cells migration with wound healing assay. As shown in Figure 1C, triptolide significantly suppressed cells migration compared to DMSO control. Meanwhile, we carried out clonogenic assay to analyze the effect of triptolide on the survivability of HEp-2 cells. Compared with the numbers and size of colonies in the control group, those in triptolide-treated groups were much fewer and smaller, suggesting that triptolide markedly suppresses survivability of HEp-2 cells (Figure 1D). These data indicate that triptolide could inhibit the proliferation, migration and survivability of laryngocarcinoma cells with high efficacy, suggesting triptolide is a potential anti-laryngocarcinoma agent. Furthermore, in order to investigate whether the effect of triptolide is tumor cell specific, we compared the efficiency of triptolide on human embryonic lung fibroblast cell (hELF), Hacat, 293 and HEp-2. The results showed that the cytotoxicity of triptolide on normal cells (except Hacat) was less than on tumor cells HEp-2 (Figure 1E). In addition, we compared the effect of triptolide between p53 wild type tumor cells or transformed cells including HEp-2, Hela and TC-1 and the p53 mutant tumor cells including PC-3, MDA-MB-468, MKN28 and FaDu. The result showed that triptolide is more effective on p53 wild type tumor cells or transformed cells compared to p53 mutant tumor cells (except MDA-MB-468) (Figure 1F). These results suggested that triptolide is a tumor specific toxic compound, and triptolide may play anti-tumor effect in a p53-dependent manner. 

### Triptolide induces apoptosis in laryngocarcinoma cells *in vitro*

**Figure 2:**
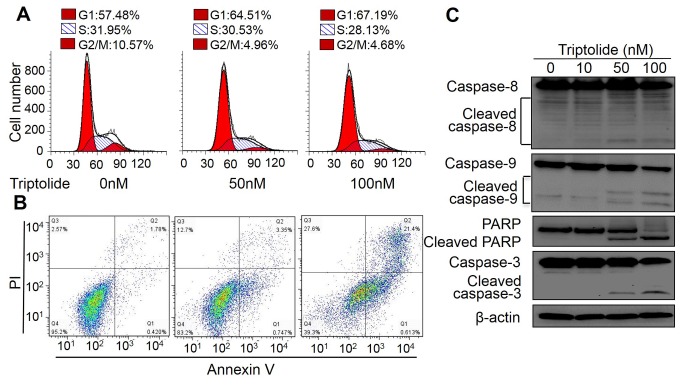
Triptolide induced apoptosis in laryngocarcinoma cell *in* *vitro*. (A) Cell cycle analysis of HEp-2 cells by Flow cytometry. After treated with various doses of triptolide, HEp-2 cells were fixed and stained with propidium iodide (PI). DNA content was detected by Flow cytometry. (B) Apoptotic analysis of HEp-2 cells by Flow cytometry. HEp-2 cells were treated with various doses of triptolide and incubated with AV-FITC (green) and PI (red). Stained cells were analyzed by Flow cytometry. Percentage of intact cells (AV-/PI−) and different stages apoptotic cells (AV+/PI−, AV+/PI+ and AV-/PI+) are presented. (C) Western blot analysis of caspase-8/9/3 proteins in triptolide-treated laryngocarcinoma cell. HEp-2 cells were treated with indicated doses of triptolide for 24 h, the procaspase-8/9/3, PARP and their cleaved products were indicated. β-actin was used as a loading control.

Since the toxicity of triptolide on cancer cells includes apoptosis induction activity, we further examined the apoptosis induction effect of triptolide in HEp-2 cells. Cells were treated with indicated doses of triptolide and subjected to analysis by flow cytometry. As shown in Figure 2A, triptolide induced cells to accumulate in sub-G1 phase in a dose dependent manner. Annexin V (AV) -FITC and PI double staining showed that triptolide induced the increase of the percentages of both early (AV+/PI-) and late apoptotic cells (AV+/PI+) (Figure 2B). These results indicated that triptolide efficiently induced laryngocarcinoma cells apoptosis. To further determine the apoptosis pathway involved, cells were treated with triptolide and subjected to the analysis of the caspase protein levels by western blot. Caspase-8 and -9 play important roles in cell apoptosis and are involved in intrinsic and extrinsic apoptotic pathways respectively. Activated caspase-8/-9 cleaves the effector caspase-3 to promote cell apoptosis. As shown in Figure 2C, the levels of cleaved caspase-8/-9/-3, were increased with triptolide treatment. Furthermore, one of the targets of caspase-3, PARP was also cleaved. These results further showed that triptolide efficiently induced laryngocarcinoma cell apoptosis through both intrinsic and extrinsic pathways.

### Triptolide enhances the anti-tumor effect of radiation in laryngocarcinoma cells *in vitro*

**Figure 3:**
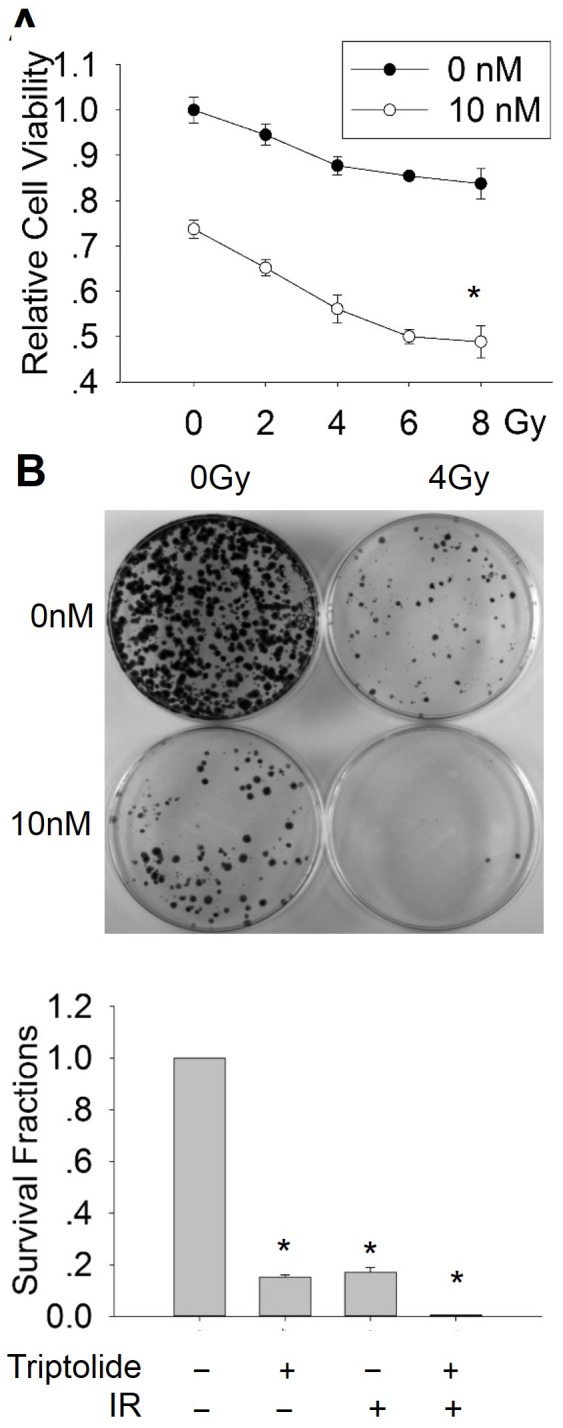
Triptolide enhanced the anti-tumor effect of radiation on laryngocarcinoma cells. (A) The combination use of triptolide with radiation showed more inhibitory effect on the HEp-2 cells viability. Cells were seeded into 96 well plates with a density of 5000 cells per well. After pro-treatment with 10nM triptolide for 5h, cells were treated with various doses of X-ray radiation. Cell viability was detected with CCK8 assay. (B) The combination use of triptolide with radiation showed more inhibitory effect on the HEp-2 cells survivability. After pro-treatment with 10nM triptolide for 5h and radiated with 4 Gy, cells were then trypsonsized and plated in 60 mm plates with a density of 1000 cells per plate. 2 to 3 weeks later, cells were fixed and stained, and the numbers of colonies were counted and the survival fractions were calculated.

Our results have shown that triptolide significantly inhibits laryngocarcinoma cell proliferation and induces cell apoptosis, which suggests triptolide may be a potential anti-laryngocarcinoma compound. Since the radiotherapy is one of the main conventional clinical therapies for cancer, we further ask whether triptolide can be used to treat laryngocarcinoma combining with radiotherapy, or if triptolide may enhance the sensitivity of laryngocarcinoma cells to radiation. HEp-2 cells were treated with triptolide combining with or without radiation, and subjected to cell viability assay and clonogenic assay. As shown in Figure 3A, the combination of radiation and triptolide induced a more pronounced anti-proliferative effect than radiation or triptolide alone. By using clonogenic survival assay, triptolide combined with radiation significantly enhanced the HEP-2 cells killing. At the dose of 4 Gy, the clonogenic ability was almost completely suppressed with the addition of triptolide (Figure 3B). These results demonstrated that the combining treatment of radiation with triptolide is more effective, suggesting triptolide may enhance the sensitivity of laryngocarcinoma cells to radiotherapy.

### Triptolide enhances p53 expression in laryngocarcinoma cells

**Figure 4:**
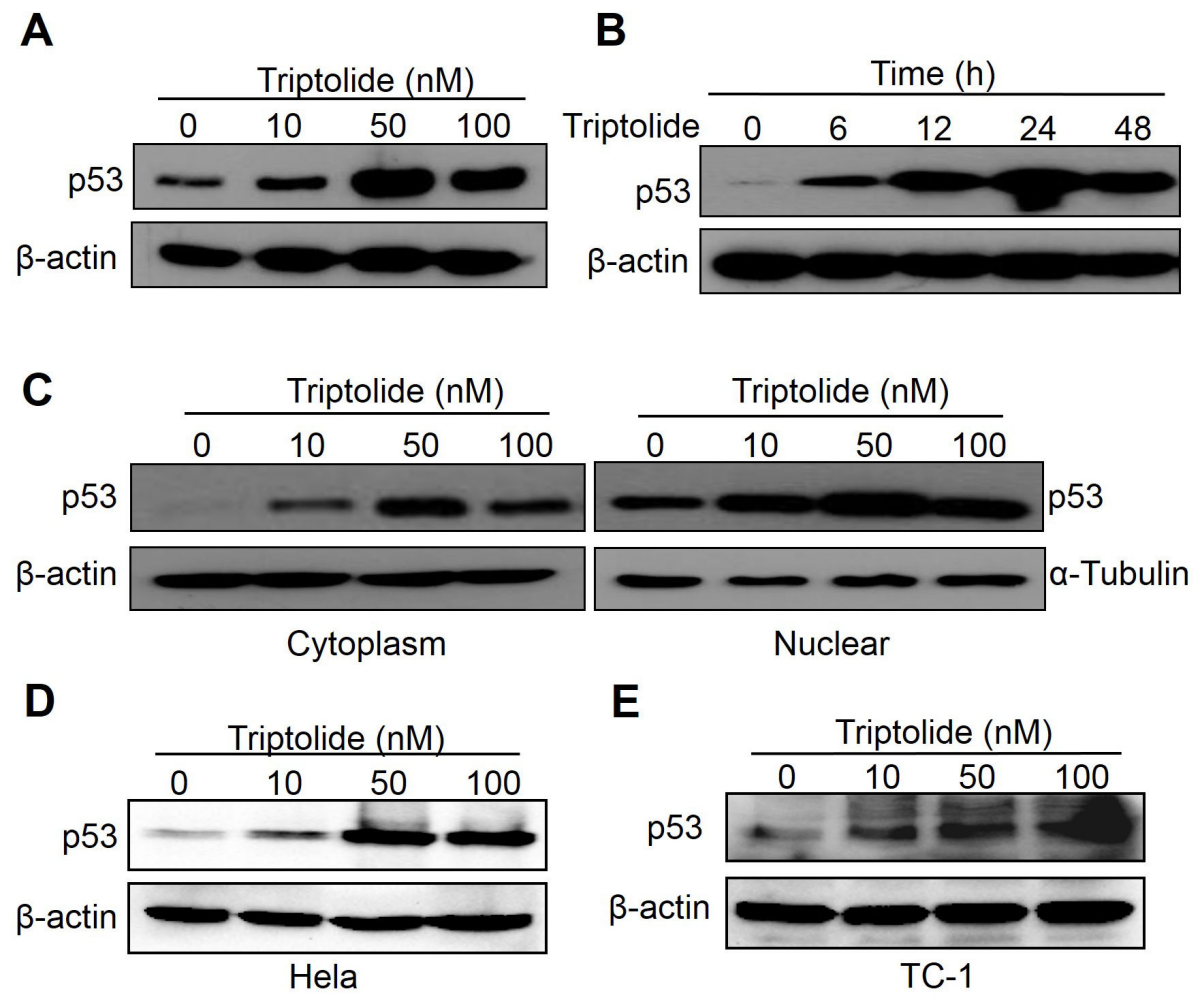
Triptolide enhanced p53 expression in laryngocarcinoma cells. (A) Triptolide enhanced p53 protein level in HEp-2 cells in a dose-dependent manner. Cells were treated with indicated doses of triptolide and analyzed by western blot. β-actin was used as a loading control. (B) Triptolide enhanced p53 protein level in HEp-2 cells in a time-dependent manner. Cells were treated with 50 nM triptolide for indicated times before Western blot analysis. (C) Triptolide induced p53 accumulation in both cell cytoplasm and nucleus. After treated with indicated doses of triptolide, the nuclear and cytosolic fraction of HEp-2 cells were extracted and subjected to analyze p53 level. β-actin and α-tubulin were used as loading control, respectively. (D) and (E) Triptolide enhanced p53 protein level in Hela and TC-1 cells in a dose-dependent manner. Cells were treated with indicated doses of triptolide and analyzed by western blot. β-actin was used as a loading control.

As the proliferation inhibition and apoptosis induction effects of triptolide in laryngocarcinoma cells have been verified, we were interested in understanding the underlying mechanism. HEp-2 cells express wild-type p53, an important tumor suppressor in cell, which expression can be enhanced in stressed cells leading to cell cycle arrest and apoptosis [8,9]. We also found that triptolide showed more cytotoxicity on tumor cells with wild-type p53 (Figure 1F). These suggest that triptolide may show anti-tumor effect in a p53-dependent manner. To analyze whether triptolide inhibits cell proliferation and induces apoptosis via p53, we examined the p53 level in HEp-2 cells treated under tritpolide treatment. As shown in Figure 4, triptolide significantly enhanced p53 expression in both dose- and time-dependent manners (Figure 4A, 4B). The multiple functions of p53 are tightly correlated with its protein level, post-modifications and subcellular localization [8,9]. In general, nuclear p53 acts as a transcription factor whereas cytoplasmic p53 plays a non-transcriptional role [29]. We therefore examined the subnuclear and cytoplasmic p53 level in HEp-2 cells treated with triptolide. The results showed that triptolide up-regulated both subnuclear and cytoplasmic p53 protein levels (Figure 4C). To confirm the effect of triptolide on p53 expression, we also examined the p53 protein level in Hela cells (HPV 18 positive) and TC-1 cells (HPV 16 E6/E7 transformed) following the treatments of various doses of triptolide. The result is similar to that in HEp-2 cells (Figure 4E, 4F). These results suggest that triptolide may inhibit HEp-2 cells proliferation and induce apoptosis through p53-related pathways.

### Triptolide increases p53 transcription and enhances p53 protein stabilization in laryngocarcinoma cells

**Figure 5:**
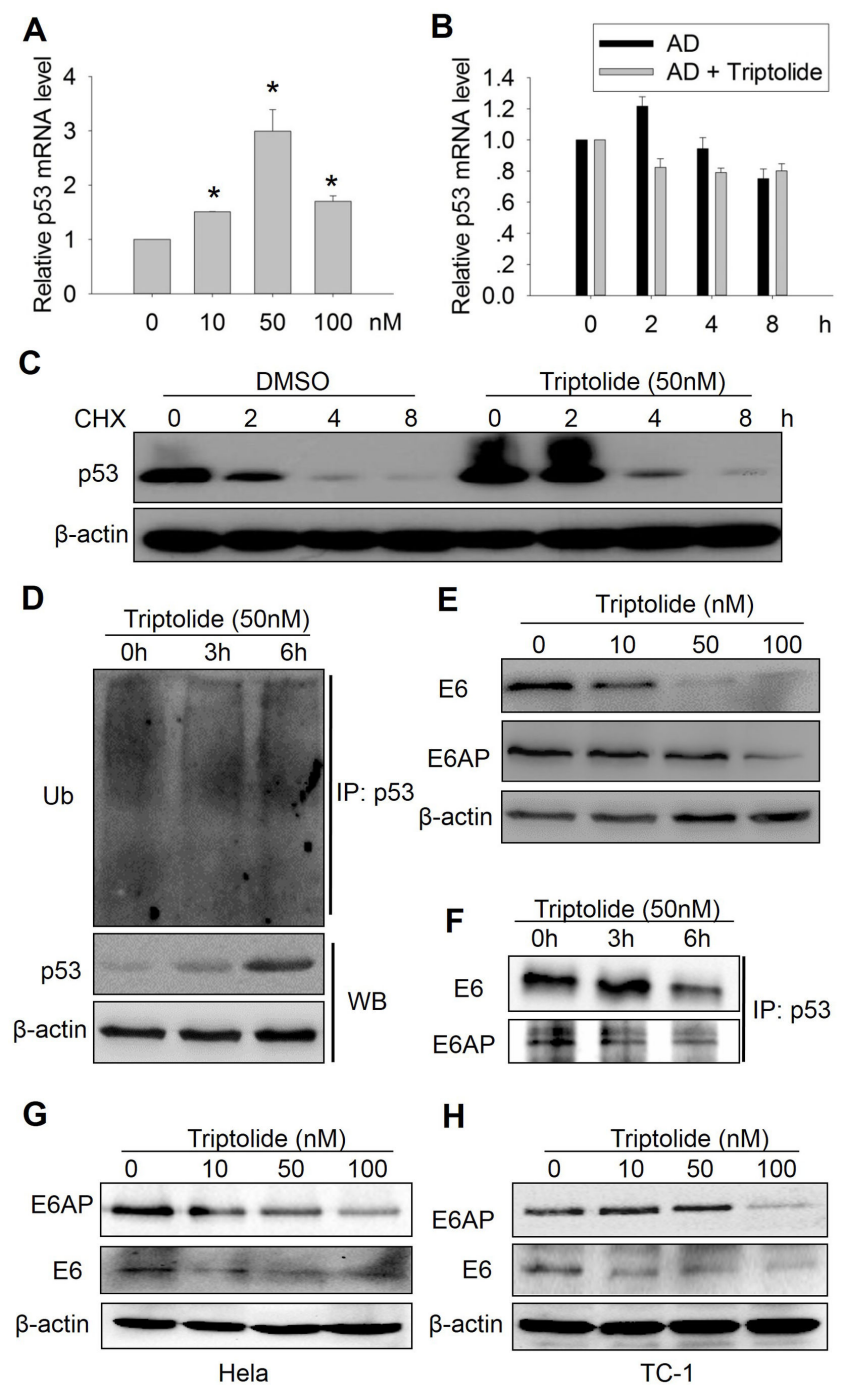
Triptolide up-regulated p53 mRNA level and enhanced p53 protein stabilization in laryngocarcinoma cells. (A) Triptolide enhanced p53 mRNA level in HEp-2 cells. Cells were treated with indicated doses of triptolide for 24h, p53 mRNA levels were determined by qRT-PCR using the specific primers. (B) Triptolide showed weak influence on the p53 mRNA stability. Cells were treated with 25μg/ml actinomycin D (AD) with or without 50 nM triptolide for indicated times, p53 and β-actin mRNA levels were determined by qRT-PCR and relative p53 mRNA level were presented. (C) Triptolide stabilized p53 protein level in HEp-2 cells. Cells were treated with 50μg/ml cycloheximide (CHX) with or without 50 nM triptolide for indicated times, p53 and β-actin protein levels were determined by western blot. (D) Effect of triptolide on p53 ubiquitination. HEp-2 cells were treated with 50 nM triptolide for indicated times, cell lysates were immunoprecipitated with p53 antibody and immunoblotted with Ub antibody. p53 and β-actin protein level were also presented. (E) Triptolide reduced E6 and E6AP expression in laryngocarcinoma cell. (F) Effect of triptolide on the interaction of p53 with E6 and E6AP. Cells were treated with indicated doses of triptolide and immunoprecipitated with p53 antibody, E6 and E6AP were immune-blotted. (G) and (H) Triptolide reduced E6 and E6AP expression in Hela and TC-1 cells.

The p53 expression is strictly regulated in cells via various mechanisms including transcription, translation, mRNA and protein stability and post-modifications [8,9]. We have found that triptolide enhances p53 expression in HEp-2 cells. To determine how triptolide up-regulates p53 protein level, we firstly measured the p53 mRNA levels in HEp-2 cells under triptolide treatments. As shown in Figure 5A, triptolide increased p53 mRNA levels. Since mRNA level is dependent on transcription and mRNA stability, we further examined the effect of triptolide on p53 mRNA stability. Actinomycin D (AD) was used to inhibit the whole mRNA production in cells, AD prevents RNA polymerase from elongating RNA chain by binding to DNA at the transcription initiation complex [30]. Cells were treated with 5μg/ml AD with or without 50 nM Triptolide for the indicated time periods and p53 mRNA level was detected. The results showed that triptolide has weak influence on the p53 mRNA stability (Figure 5B), suggesting induction of p53 transcription rather than p53 mRNA stability may contribute to up-regulation of p53 by triptolide.

In addition to the transcriptional regulation, p53 level is mainly controlled by ubiquitination-mediated degradation [8]. So we also examined the p53 protein stability under triptolide treatment. Another compound cycloheximide (CHX) was used to inhibit total protein synthesis in cells. Cells were treated with 50μg/mL CHX with or without 50 nM triptolide for the indicated time periods, and p53 protein level was detected. As shown in Figure 5C, triptolide also enhanced the p53 protein stability. Since p53 protein level is strictly controlled by ubiquitination -mediated degradation, we examined p53 ubiquitination following triptolide treatment. Total endogenous p53 was immunoprecipitated and ub-p53 level was analyzed. The results showed that the ubiquitinated p53 level was decreased under triptolide treatment, despite that the total p53 level was increased (Figure 5D). These results indicated that triptolide enhanced p53 expression partially through inhibiting p53 degradation. 

In HPV negative cells, p53 protein stability is mainly controlled by the E3 ligase MDM2 mediated p53 ubiquitination and degradation by the proteasome [31]. However, in HPV-positive HEp-2 cells, the effect of MDM2 is replaced by a viral protein E6 and its associated protein, an ubiquitin E3 ligase E6AP. The E6/E6AP complex binds to and ubiquitinates p53, subsequently leading to p53 proteasomal degradation [32]. To examine whether E6 and/or E6AP are involved in the inhibitory effect of triptolide on the ubiquitination-mediated p53 degradation, we analyzed the E6 and E6AP levels in HEp-2 cells under triptolide treatment. As shown in Figure 5E, triptolide markedly suppressed both E6 and E6AP expression in a dose-dependent manner. Furthermore, we analyzed the interaction between p53 and E6 or E6AP by IP following triptolide treatment. The result showed that both p53-E6 and p53-E6AP interactions were inhibited (Figure 5F). Similarly, expression of E6 and E6AP in Hela and TC-1 cells were also inhibited under triptolide treatment (Figure 5G, 5H).These results indicated that triptolide may enhance p53 expression partially through inhibition of E6/E6AP mediated p53 ubiquitination and degradation. 

### Effect of Triptolide on the functions of p53 in laryngocarcinoma cells

**Figure 6:**
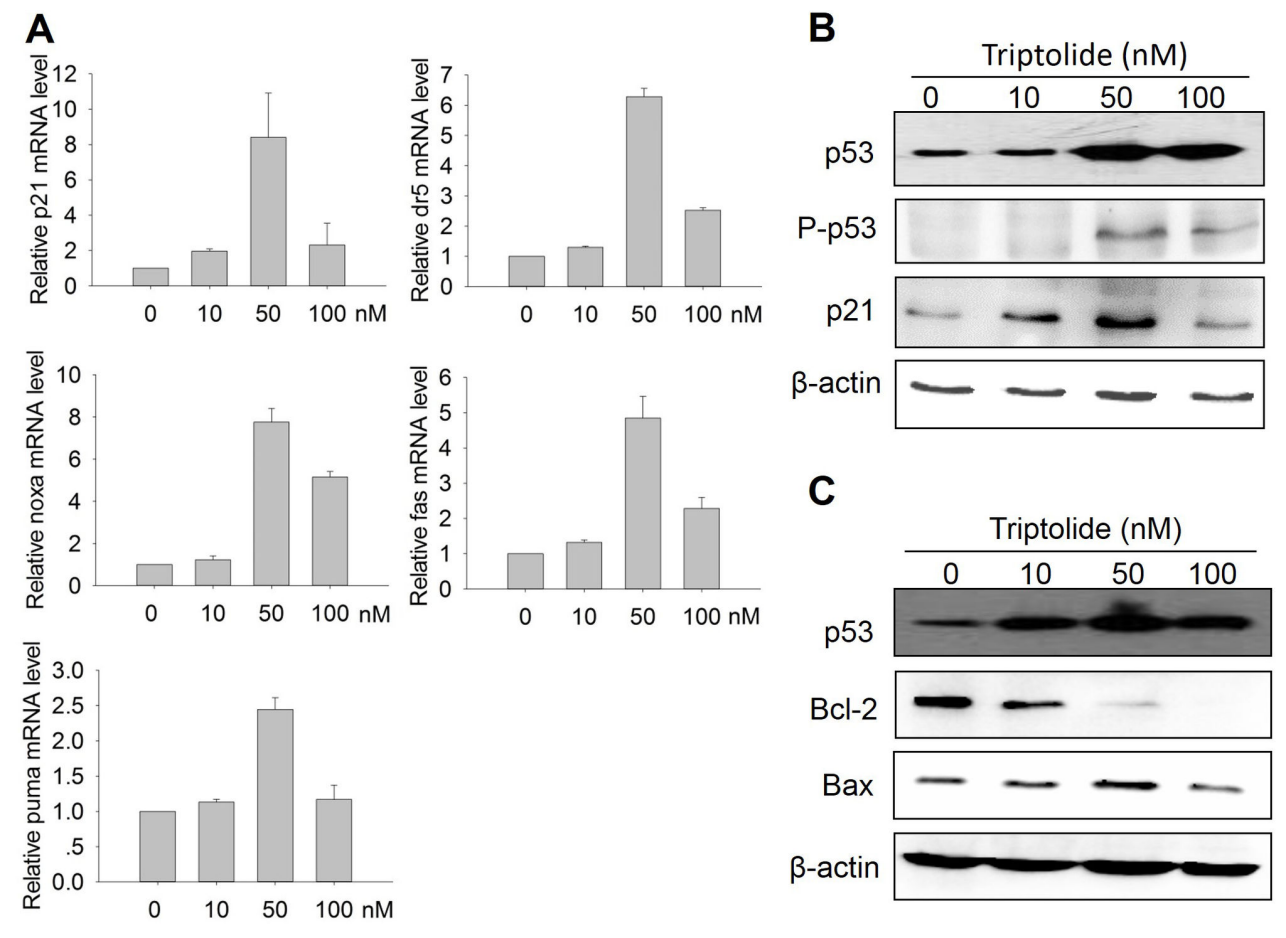
Effect of Triptolide on the p53 function in laryngocarcinoma cells. (A) Effect of triptolide on the transcription of p53 target genes. Total mRNA was extracted from HEp-2 cells treated with various doses triptolide for 24h. The mRNA levels of several p53 target genes, i.e. p21, fas, dr5, noxa and puma, were analyzed by real-time PCR. (B) Effect of triptolide on the transcriptional function of p53. HEp-2 cells were treated with triptolide and subjected to analyze p53, p-p53 (S15) and p21 expression by western blot. (C) Effect of triptolide on the Bcl-2 family proteins expression. HEp-2 cells treated with triptolide were collected to detect the p53, Bcl-2 and Bax expression.

As one of the most important tumor suppressors in the cell, p53 has multiple functions [8]. Being a transcription factor, p53 binds to the response element on down-stream target genes and activates or represses these genes expression, leading to cell cycle arrest, DNA damage repair and initiation of apoptosis [8]. On the other side, p53 links directly with many cell pathways to induce DNA repair or apoptosis [9]. To examine the effect of triptolide on p53 functions, we firstly examined several p53 target genes expression by qPCR, such as p21, fas, dr5, puma and noxa, which are important pro-apoptotic genes positively regulated by p53. The results showed that mRNA levels of these p53 target genes were up-regulated (Figure 6A), indicating that triptolide also enhances p53 transcriptional activity. Furthermore, since the p53 transcriptional function is also regulated by post translational modifications, especially phosphorylation and acetylation, we analyzed the p53 Ser15 phosphorylation, which is critical for p53-dependent transactivation [33]. As shown in Figure 6B, following treatment with increasing concentrations of triptolide, the p-p53 (Ser15) level was up-regulated, and the key p53 target protein p21 level was also increased. We also checked the level of the important anti-apoptotic protein Bcl-2 which is negatively regulated by p53. Contrast to the increased expression of p53, the anti-apoptosis protein Bcl-2 was decreased by triptolide in a dose-dependent manner, while the pro-apoptosis protein Bax level was increased (Figure 6C). These results demonstrated that triptolide not only enhances p53 expression, but also promotes p53 functions to induce cell cycle arrest and apoptosis.

### Triptolide induces DNA damage in laryngocarcinoma cells

**Figure 7:**
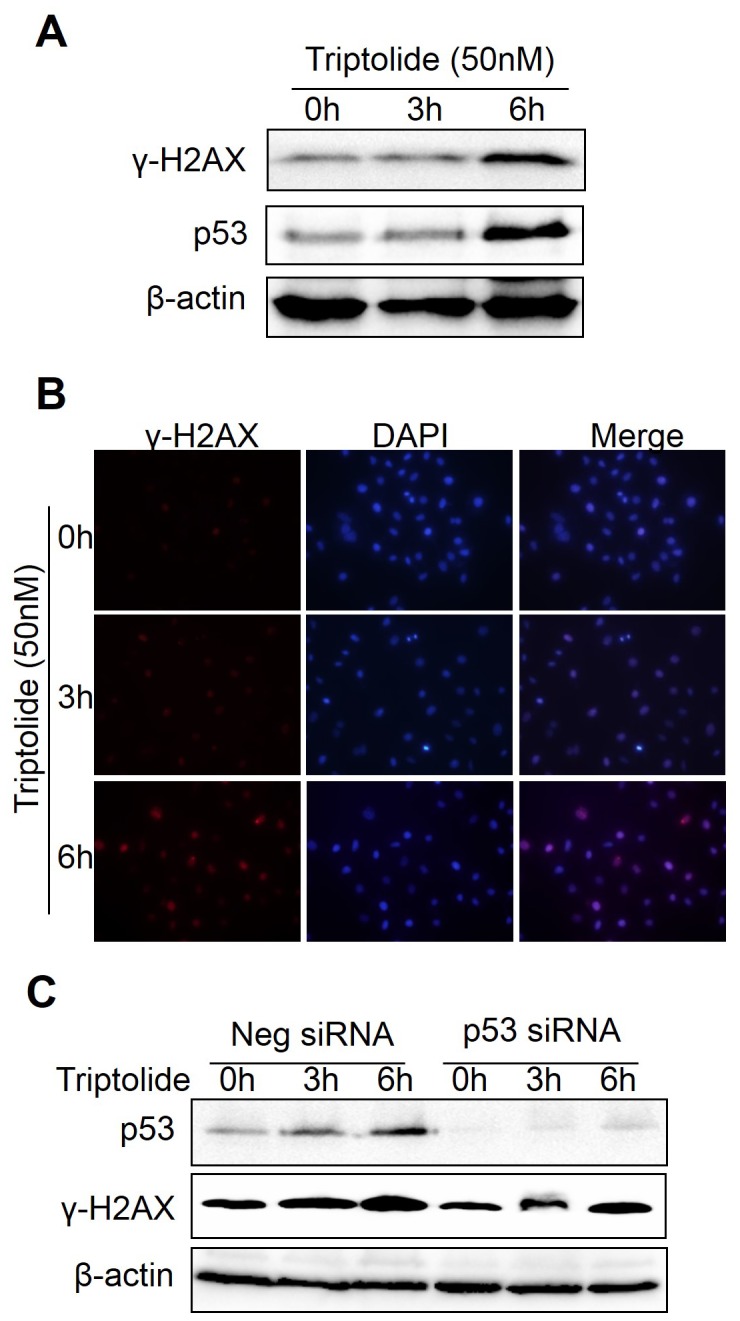
Triptolide induced DNA damage in laryngocarcinoma cells. (A) Effect of triptolide on the DNA damage. HEp-2 cells were treated with 50nM triptolide for indicated times and subjected to detect the γ-H2AX and p53 levels by western blot. (B) The IF results of γ-H2AX expression in tritpolide treated HEp-2 cells. Cells were fixed, incubated with rabbit anti-γ-H2AX primary antibody and anti-rabbit secondary antibody conjugated with Alex Flour 555 (Red) and stained with DAPI (Blue). Cell images were captured with fluorescence microscopy. (C) Effect of p53 knockdown on the induction of DNA damage by triptolide. HEp-2 cells were transfected with p53 siRNA or negative siRNA oligonucleotide for 24h, then treated with 50nM triptolide for indicated times. Cells were collected to detect the γ-H2AX and p53 levels by western blot.

As an important senser of various harmful genotoxic stresses, p53 is activated in stressed cells to induce several responses to protect normal cell or inhibit the survivability of malignant cell [8,9]. The anti-tumor effect of triptolide attributes to its cell toxicity, mainly presenting as apoptosis induction. We hypothesized that triptolide may induce DNA damage to impel p53 expression and functions. We therefore examined the effect of triptolide treatment on the level of γ-H2AX (phosphorylated histone H2AX on serine 139), a sensitive DNA damage marker specifically induced by DNA Double-Strand Breaks (DSB) [34]. As shown in Figure 7A, γ-H2AX level was up-regulated by triptolide after a short time of treatment, similar to that of p53. We further examined the γ-H2AX using immunofluorescence. The results showed that the γ-H2AX signal was gradually increased following triptolide treatment (Figure 7B). In addition, we investigated the effect of triptolide on DNA damage after p53 knockdown. However, γ-H2AX expression was still increasing significantly in a time dependent manner after p53 knockdown followed by triptolide treatment, which indicated that p53 knockdown do not inhibit triptolide-induced DNA damage. (Figure 7C). It might be possible that triptolide-triggered DNA damage is an inducer of p53 accumulation rather than an outcome of p53-induced apoptosis. However, the detailed underlying mechanism still needs more study. These results suggest that triptolide may trigger DNA damage-induced p53 accumulation, leading to HEp-2 cells apoptosis.

### Role of p53 and caspases in the anti-tumor effect of triptolide

**Figure 8:**
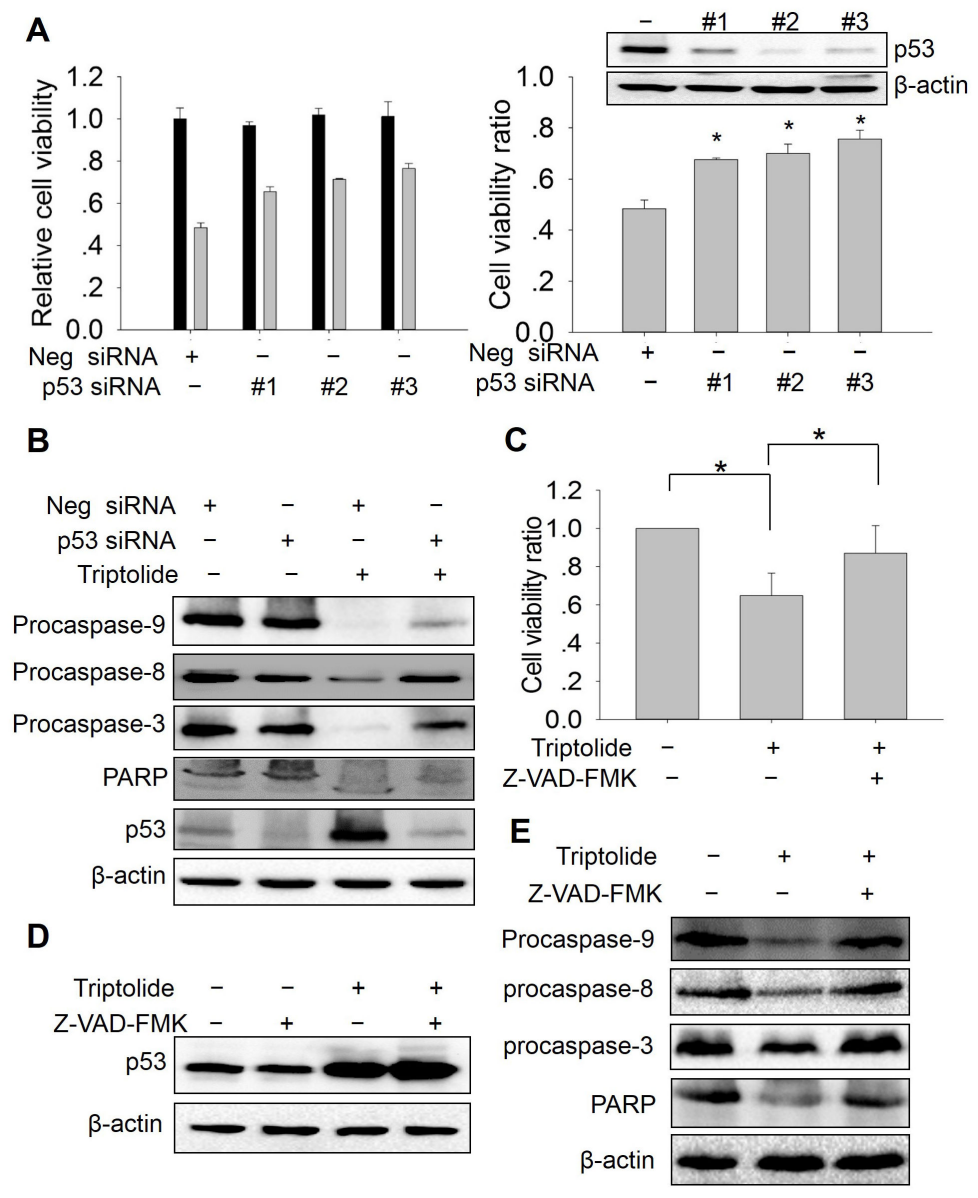
Role of p53 and caspases in the anti-tumor effect of triptolide. (A) Effect of p53 knockdown on cell toxicity of triptolide. HEp-2 cells were transfected with p53 siRNA or negative siRNA oligonucleotide for 24h, then treated with 50nM triptolide for additional 24h. Cell viability was measured using CCK-8 assay. Left chart showed the relative cell viability. Right chart showed the cell viability ratio. (B) Effect of p53 knockdown on the induction of caspases and PARP cleavage by triptolide. After transfected with p53 siRNA or negative siRNA oligonucleotide and treated with 50nM triptolide, HEp-2 cells were collected to detect caspase-9/8/3 and PARP by western blot. β-actin was used as internal control. (C) Effect of caspases inhibitor on the cell toxicity of triptolide. After treatment with 100μM caspases inhibitor Z-VAD-FMK for 1h and 50nM triptolide for additional 24h, HEp-2 cells viability was measured using CCK-8 assay and the cell viability ratio were calculated. (D) Effect of caspases inhibitor on p53 expression. After treatment with 100μM Z-VAD-FMK and 50nM triptolide, HEp-2 cells were collected to detect p53 protein level by western blot. (E) Effect of caspases inhibitor on the caspase-9/8/3 and PARP cleavage. After treatment with 100μM Z-VAD-FMK and 50nM triptolide, HEp-2 cells were collected to detect caspase-9/8/3 and PARP by western blot. β-actin was used as internal control. The asterisks indicates P <0.05.

Triptolide inhibited HEp-2 cells proliferation, induced cell apoptosis, and enhanced p53 expression and functions. To examine the relationship between triptolide induced p53 up-regulation and cell toxicity on laryngocarcinoma cells, we analyzed the effect of p53 knockdown on triptolide anti-tumor activity. Cells were transfected with p53 siRNA for 24h and treated with 50nM triptolide for another 24h, cell viability was measured using CCK-8 reagent. As shown in Figure 8A, compared to control group, the cell viability of p53 knockdown groups presented varying degrees of increase under triptolide treatment (Figure 8A left chart). We further calculated the cell viability ratio of each groups. The result showed that p53 knockdown significantly promotes the cell viability upon triptolide treatment, especially the #2 and #3 p53 siRNA (Figure 8A right chart). These data indicated that p53 plays an important role in the effect of triptolide in HEp-2 cells. In addition, we investigated the effect of triptolide on caspase 3/8/9 and PARP cleavage after p53 knockdown. The data show that knockdown of p53 reduces caspase 3/8/9 and PARP cleavage (Figure 8B). These results together suggest that triptolide may act in a p53-dependent manner. Furthermore, we detected the effect of caspases inhibitor on triptolide cytotoxicity. HEp-2 cells were treated with 100 μM Z-VAD-FMK with or without 50 nM Triptolide for 24h and subjected to analysis of the cell viability. The result showed that the cell viability of the group co-treated with Z-VAD-FMK and triptolide was markedly increased compared to the triptolide alone treated group (Figure 8C). We also evaluated the p53, caspase-8/-9/-3 and PARP of each group. The results showed that Z-VAD-FMK does not affect p53 expression (Figure 8D), but inhibits the caspases cleavage induced by triptolide. (Figure 8E). Taken together, these results demonstrated that inhibiting caspases activity could suppress the cytotoxicity of triptolide, suggesting that apoptosis induction is the main action mode by which triptolide elicit its anti-tumor effect in HEp-2 cells.

## Discussion

As the main active compound of *Tripterygium wilfordii* Hook *F*., triptolide shows potent anti-tumor effects. Triptolide inhibits the growth of many types of cancer cells [14], with IC_50_ at nanomolar levels in all 60 cancer cell lines from the US national Cancer Institute [28]. But the effect of triptolide on laryngocarcinoma cells has not been well characterized. In this study, we demonstrated that triptolide markedly suppressed laryngocarcinoma cells HEp-2 growth in a dose-dependent manner, and with the IC_50_ value of 39.5 nM. Furthermore, we also found that triptolide significantly inhibited HEp-2 cells migration and survivability. Johnson et al. [35] found that triptolide inhibits proliferation and migration of colon cancer cells through inhibition of multiple cytokine receptors. Tan et al. [36] reported that triptolide reduces breast cancer cells MCF-7 adhesion and survival via induction of FAK cleavage. These studies suggest that triptolide can suppress cancer metastasis in addition to cell growth inhibition. Radiotherapy is one of main clinical therapies for treatment of laryngocarcinoma. Nowadays radiotherapy combined with other therapy is used frequently to treat cancers, and shows more curative effect. Some chemical drugs were found to enhance the radiosensitivity of tumor cells. In this study, we found that combination of triptolide with radiation showed more effective anti-tumor activity compared to the treatment with triptolide or radiation alone. Wang et al [37] also found that triptolide sensitized pancreatic cancer cells to radiation therapy. These findings suggest that triptolide may be a potent radiosensitizer in laryngocarcinoma therapy. Taken together, tripotlide is a potential drug for laryngocarcinoma therapy based on its potent anti-tumor effect.

Apart from inhibiting cancer cells proliferation, the anti-tumor effect of triptolide can also be attributed to induction of cellular apoptosis. Triptolide was reported to induce apoptosis in many types of cancer cells [38]. In the present study, we also found that triptolide induces laryngocarcinoma cells cycle arrest and apoptosis. Generally, apoptosis is induced through two pathways: intrinsic and extrinsic pathways. In the two pathways, the initiation caspases caspase-9 and caspase-8 are activated first, which further induces the activation of the effector caspase-3. Activated caspase-3 cleaves target proteins including PARP and induces cell apoptosis. It was reported that triptolide mediates apoptosis through both pathways mentioned above. Triptolide was demonstrated to induce apoptosis in cervical cancer cells [39] and malignant schwannoma cells [40] accompanied with caspase-8/9/3 and PARP cleavage, indicating the activation of the two apoptotic pathways. Our previous study showed that triptolide induces prostate cancer cells apoptosis through both intrinsic and extrinsic pathways [24]. However, Lu et al. [41] reported that triptolide strongly induces caspase-9 and PARP cleavages in MCF-7 cells, but fails to activate caspase-8. Carter et al. [42] found that triptolide fails to induce apoptosis in caspase-9 knock-out leukemic cell lines, but remains effective on caspase-8 deficient cells. Triptolide was also reported to sensitize resistant cholangiocarcinoma cells to TRAIL-induced apoptosis through extrinsic pathways [43]. These studies suggest that the mechanism of triptolide-induced apoptosis varies among different types of cancers. In the present paper, our results show that capsase-9/-8/-3 and PARP are cleaved in triptolide treated HEp-2 cells, suggesting that triptolide induces HEp-2 cells apoptosis via the two pathways. Furthermore, we found that triptolide increases the mRNA level of apoptosis related genes in treated cells, including fas, dr5, noxa and puma. fas and dr5 encode death receptors, which initiate extrinsic pathway after binding with their respective specific ligands. noxa and puma are intrinsic pathway-related pro-apoptosis proteins, which associate with Bcl-2 family proteins to change the mitochondria membrane permeability, resulting in cytochrome C release and intrinsic pathway activation. We also found triptolide decreases the level of the key anti-apoptosis protein Bcl-2. On the contrary, the pro-apoptosis protein Bax was induced. These results further demonstrated that triptolide induces HEp-2 cells apoptosis via both intrinsic and extrinsic pathways. Moreover, caspases inhibitor Z-VAD-FMK treatment markedly suppressed the cytotoxicity of triptolide in HEp-2 cells, indicating the anti-tumor effect of triptolide is mainly dependent on its ability to induce apoptosis. 

The mechanism of triptolide anti-tumor effect was further studied. Triptolide was demonstrated to down-regulate many genes expression, which was attributed to its transcription inhibition effect. Triptolide was reported to induce the RNA pol II transcription inhibition through binding to the transcription factor TFIIH subunit XPB [28] and inducing another RNA pol II subunit Rpb1 degradation [44,45]. Meanwhile, several genes were found to be up-regulated in cells following triptolide treatment [14]. In this study, we found that triptolide enhances p53 expression in HEp-2 cells in dose-dependent and time-dependent manners. p53 is one of the most important guardian of normal cells. Activated by genotoxic stresses, p53 mediates cell cycle arrest, DNA repair, senescence and apoptosis. So, the effect of triptolide on HEp-2 cells may be due to p53 activation. Triptolide was demonstrated to inhibit cell growth and induce apoptosis in gastric cancer cells with wild-type p53, whereas no significant effect was observed on cells with mutant p53 [46]. Kiviharju et al. [47] reported that high concentrations of triptolide induced apoptosis in apoptotic-resistant primary cultured human prostatic epithelial cells are associated with nuclear accumulation of p53. Vispé et al. [48] also found triptolide induces persistent p53 accumulation in adenocarcinomic human alveolar basal epithelial cells A549. Hu et al. [49] found that triptolide sensitizes TRAIL-mediated apoptosis in prostate cancer through induction of p53 accumulation and thereby up-regulating dr5 expression. Moreover, triptolide was demonstrated to suppress p53-dependent p21 induction and modulates the p53 transcriptional activity in a cancer-specific manner, leading to enhancement of CDDP-induced apoptosis in urothelial cancer cells with wild-type p53, but not in those with mutant p53 or normal human urothelium cells [50]. These studies indicate that triptolide mediates cellular apoptosis in a p53 dependent manner in cells with wild-type p53. Triptolide also was demonstrated to show anti-tumor effect in cells in a p53-independent manner. Hang et al. reported that triptolide induces apoptosis in a subgroup of acute lymphoblastic leukemia (ALL) cells by inhibiting MDM2 expression, leading to p53 increase without activation [51]. However, triptolide shows more effective anti-cancer activity in cells with WT p53 compared to those with mutant/null p53. Although triptolide induces apoptosis in all cells with wild-type or mutant p53, the IC_50_ of triptolide in cells with wild-type p53 are much lower than cells with mutant /null p53 [51]. Our previous study also showed that triptolide induces apoptosis in both LNCap (p53 intact) and PC-3 (p53 mutant) Prostate cancer cells lines regardless of their p53 status, but triptolide was more effective in LNCap cells [24]. These studies demonstrate that triptolide displays higher effect in cells with wild-type p53. 

p53 functions through transcription-dependent and transcription-independent pathways [8]. Activated p53 translocates to the nucleus and binds to the promoter of its target genes to play its transcriptional role, either transactivation or suppression [8]. We found p53 accumulation in both nucleus and cytoplasm, suggesting both of the p53 mechanisms of action are activated. p53 Ser15 phosphorylation level was also enhanced by triptolide, so was several key target genes positively regulated by p53. In addition, knockdown of p53 significantly inhibited caspase 3/8/9 and PARP cleavage, and suppressed triptolide anti-cancer effect in HEp-2 cells. Taken together, these results indicate that triptolide inhibits HEp-2 cells proliferation and induces apoptosis in a p53-dependent manner. 

Generally, p53 protein level in normal cells is strictly controlled and kept at low steady-state level, but p53 could be induced and activated quickly when cells suffer genotoxic stresses. Several reports showed that the p53 response to genotoxic stresses is partially regulated by transcription. Hellin et al. [52] reported that anthracycline daunomycin induces p53 transcription partially via NF-kappaB transcriptional activity. Wang et al. [53] found that several DNA-damaging chemical agents induce p53 mRNA level increase. Except for transcription regulation, p53 mRNA stability is also strictly controlled [54]. The double-strand RNA binding zinc finger protein Wig-1 promotes p53 mRNA stability through binding to an AU-rich element in the 3’-UTR of p53 mRNA [55].The ribosomal protein RPL26 enhances p53 mRNA translation through binding to the 5′UTR of p53 mRNA [56,57]. In the current study, we found that triptolide induces p53 transcription activation, but fails to stabilize p53 mRNA. We further found that the marker of DNA Double-strand breaks (DSB) γ-H2AX level was induced by triptolide in HEp-2 cells correlating with p53 expression increase, indicating triptolide may induce DNA damage. Chueh et al. [58] also reported that triptolide induces DNA damage in human melanoma A375.S2 cells and reduces DNA repair genes expression. Taken together, triptolide may enhance p53 expression through DNA damage-induced p53 transcription. Moreover, the rapid protein turnover of p53 in normal cells is mainly regulated by MDM2-mediated ubiquitination and degradation. The induction and activation of p53 in stressed cells result from p53-MDM2 interaction disruption and MDM2 E3 ligase activity inhibition. However, in HEp-2 cells, MDM2 is substituted by E6/E6AP. E6 associates with E6AP to mediate p53 ubiquitination and degradation, contributing to laryngocarcinoma formation. On the other hand, they are the potential targets for laryngocarcinoma therapy [59,60]. Our results showed that triptolide suppresses both E6 and E6AP expression, and inhibits the interaction between p53 with E6 and E6AP, resulting in the decrease of the ubiquitinated p53 level. This indicates triptolide partially enhances p53 expression through suppression of E6 and E6AP expression, thus inhibiting p53 ubiquitin-mediated degradation. Altogether, our finding indicates that triptolide effects on HEp-2 cells in a p53-dependent manner by inducing p53 transcription and increasing its stability. 

**Figure 9:**
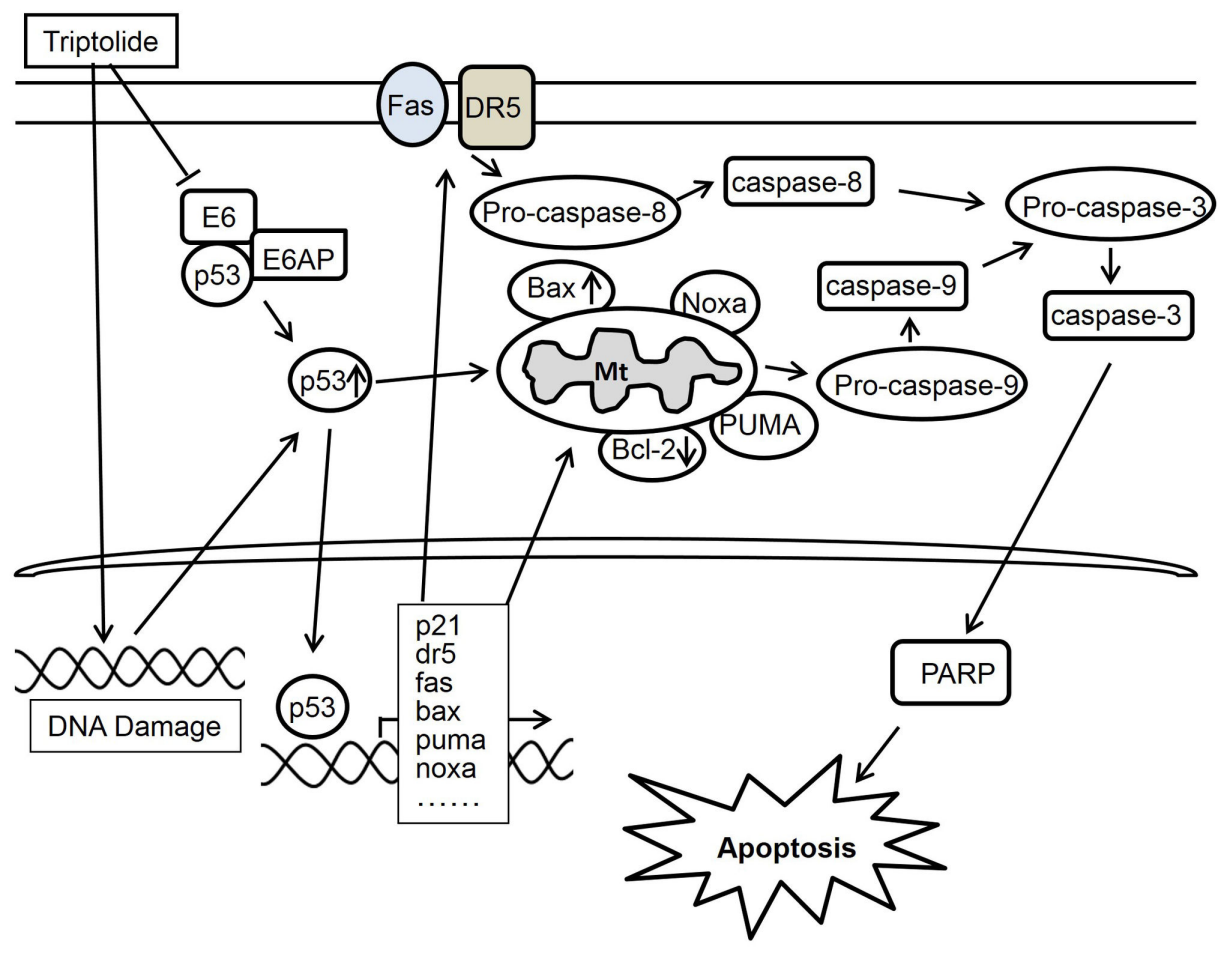
Schematic diagram of hypothetic mechanisms underlying triptolide induction of apoptosis in laryngocarcinoma cells. Triptolide causes DNA damage to induce p53 transcription. Meanwhile triptolide enhances p53 protein stability by suppression of the E6-mediated p53 ubiquitinylation-mediated degradation. Elevated p53 initiates cell apoptosis through death receptors pathways and mitochondrial pathway via activating transcription and non-transcriptional functions (see Discussion for detail).

Triptolide was demonstrated to inhibit the proliferation of many types of cancer cells and induce apoptosis through the targeting of different genes or proteins. Our study showed that triptolide has potent anti-tumor effect in laryngocarcinoma cells HEp-2. Based on our results and others studies, we propose that triptolide effects on HEp-2 cells in a p53-dependent manner. As shown in Figure 9, triptolide triggers DNA damage to induce p53 transcription. Meanwhile, triptolide inhibits the E6/E6AP mediated p53 ubiquitination and degradation to stabilize p53. As a result, p53 is induced and activated in HEp-2 cells under triptolide treatment. Activated p53 regulates down-stream target genes to mediate cell cycle arrest and apoptosis through transcription-dependent pathway. Among the p53 targets, p21 is a key one which mediates cell cycle arrest to facilitate DNA repair. Both dr5 and fas encode death receptors and mediate extrinsic apoptotic pathway. Decreased expression of anti-apoptotic protein Bcl-2 and increased expression of pro-apoptotic genes bax, puma and noxa facilitate intrinsic apoptotic pathway. Simultaneously, activated p53 further promotes apoptosis via transcription- independent pathway. Initiated intrinsic and extrinsic pathways activate caspase-8 and -9 respectively, leading to caspase-3 cleavage and cell apoptosis. 

In conclusion, our study demonstrated that triptolide displays anti-cancer effect in laryngocarcinoma cells HEp-2 as evidenced by inhibition of cells proliferation, migration, survivability, and induction of apoptosis, suggesting that triptolide is a potential drug for laryngocarcinoma therapy. The antitumor effect of triptolide is resulted from its ability to induce p53 activition via induction of DNA damage and suppression of E6/E6AP-mediated p53 ubiquitination and degradation. 

## Materials and Methods

### Reagents

Triptolide (> 98%) was purchased from π-π technologies, Inc (Shenzhen, China) and dissolved with DMSO at a stock concentration of 50 mM. Trypan blue solution and crystal violet were from Sigma-Aldrich. Complete Protease Inhibitor Cocktail Tablets (4693116001) was got from Roche. SuperSignal West Pico Chemiluminescent Substrate (34080) was purchased from Thermo Scientific. Lipofectamin 2000 was from Invitrogen. Actinomycin D, cycloheximide, caspase inhibitor Z-VAD-FMK, 4% Paraformaldehyde solution, Annexin V-FITC Apoptosis Assay Kit (C1063), Nuclear and Cytoplasmic Protein Extraction Kit (P0027), RIPA lysis buffer (P0013B) and NP-40 lysis buffer (P0013F) were from Beyotime Co. (Jiangsu, China). RNAiso Plus (9109), PrimeScript™ RT Master Mix (Perfect Real Time) (RR036A) and SYBR^®^ Premix Ex Taq^TM^ II (Tli RnaseH Plus, DRR820) were from TaKaRa (Dalian, China). Antibodies for caspase-8 (AC056), caspase-9 (AC062), caspase-3 (AC031), PARP (AP102), p-p53 (S15) (AP068), p21 (AP021), α-tubulin (AT819), and Alexa Fluor 555-labeled goat anti-rabbit IgG were from Beyotime Co. (Jiangsu, China). Antibodies for p53 (sc-126), E6 (sc-460), Ub (sc-9133), E6AP (sc-25509), and Protein A/G plus-Agarose (sc-2003) were from Santa Cruz Biotechnology. Antibody for γ-H2AX (9718), Bcl-2 (2870) and Bax (2772) were from Cell Signaling Technology. Antibody for β-actin (CW0096A), goat anti-rabbit and anti-mouse secondary antibodies conjugated with horseradish peroxidase (HRP) were from CWBIO (Beijing, China). 

### Small interfering RNAs and oligonucleotides

Three p53 siRNAs and one Non-targeting siRNA were purchased from GenePharma Co. (Shanghai, China). The target sequences of p53 siRNAs are: 5′- UGGUUCACUGAAGACCCAGTT-3′, 5′- CCACCAUCCACUACAACUATT-3′ and 5′- GACUCCAGUGGUAAUCUACTT-3′, while the sequence of Non-targeting control siRNA is 5′- UUCUCCGAACGUGUCACGUTT-3′. The primer oligonucleotides used in Real-time PCR were synthetized by Invitrogen, which sequences are as follows: p53, 5’-TCAACAAGATGTTTTGCCAACTG-3’ and 5’- ATGTGCTGTGACTGCTTGTAGATG-3’; and p21, 5’-CTGGAGACTCTCAG GGTCGAA-3’ and 5’- GGCGTTTGGAGTGGTAGAAATC-3’; and dr5, 5’- TGGTT CCAGCAAATGAAGGTG-3’ and 5’- CCGCTGCCTCAGCTTTAGC-3’; and noxa, 5’- GTGTTCCTGTTGGGCGTTAC-3’ and 5’- GGAGCATTTTCCGAACCTT-3’; and fas, 5’- AGCTTGGTCTAGAGTGAAAA-3’ and 5’- GAGGCAGAATCAT GAG ATAT-3’; and puma, 5’- GACCTCAACGCACAGTACGAG-3’ and 5’- AGGAGT CCCATGATGAGA TTGT-3’; and β-actin, 5’- AATGTCGCGGAGGACTTTGAT-3’ and 5’- AGGATGG CAAGG GACTTCCTG-3’.

### Cell culture and transient transfection

HEp-2, Hela, TC-1, PC-3, MKN28 and 293 cells were purchased from the Institute of Basic Medical Sciences, Chinese Academy of Medical Sciences. FaDu and MDA-MB-468 were purchased from the cell bank of the Chinese Academy of Science. Human embryonic lung fibroblast cells (hELF) were kindly provided by Dr. Wenwen Jia from the Tongji University (Shanghai, China). Hacat cells were a gift from Dr. Lei Lei from Xijing hospital (Shaanxi, China). HEp-2, Hela, 293, Hacat, MDA-MB-468 and hELF cells were cultured in DMEM medium (GIBCO). TC-1, PC-3 and MKN28 cells were cultured in RPMI 1640 medium (GIBCO). FaDu cells were cultured in MEM medium (GIBCO). All these medium were supplemented with 10% fetal bovine serum (FBS), 100 units/ml of penicillin and streptomycin. Cells incubated at 37°C with 5% CO_2_. Sub-confluent cells with exponential growth were used in all experiments. Transfections were carried out by using Lipofectamine 2000 according to the manufacturer’s instructions. 

### Cell proliferation assay

1×10^5^ HEp-2 cells per well were plated in 24-wells plates until attachment. Then cells were treated with various doses of triptolide, DMSO was used as negative control. Cells were trypsinized and stained with trypan blue dye, and viable cells were counted using cell counting chamber every 24h for a total of 7 days. Viable cell numbers of each group were collected and used to plot the cell growth curves. 

### Cell viability assay

5000 cells per well were plated in 96-wells plate, cultured until attachment, then treated with various doses of triptolide, using DMSO as negative control and culture medium as blank control. 24h or 48h after treatment, 10μl CCK-8 solution per well was added and the plate was incubated for 1h at 37°C. The absorbance of each well was measured on an M200pro Multimode Plate Reader (Tecan, Switzerland) at 450 nm and 650 nm. Each treatment was performed in triplicate and experiments were repeated over 3 times. IC_50_ was calculated with GraphPad Prism 5.04 (GraphPad Software, Inc.) using a sigmoidal dose-response nonlinear regression analysis. 

### Wound healing assay

HEp-2 cells were plated in 60 mM dishes until confluence. After a 3h cells pre-treatment with 50μM mytomicin C, wounds were created by scratching cell sheets with a sterile 200μl pipette tip. The culture medium was replaced with fresh medium containing either DMSO or 10nM Triptolide. The pictures of a specific position on the scratched areas were taken by an inverted microscope (Leica, Germany) using a 10 × objective every 24h. The wound widths were measured and the relative wound widths were calculated. Data are shown as mean± SD of 3 independent experiments. 

### Clonogenic assay

Clonogenic assay was carried out according to the reported protocol [61]. HEp-2 cells were trypsinized and diluted to a density of 1 × 10^4^ cells/ml. 1000 cells were plated in 60mm dishes and cultured in medium containing DMSO or 10nM triptolide. Each treatment was performed in triplicate. 2 to 3 weeks later, cell clones were fixed with 4% paraformaldehyde solution and stained with 0.1% crystal violet. Pictures of stained cell clones on plates with different treatments were captured using ChemiDoc XRS+ imaging system (Bio-Rad, USA). The surviving fraction (SF) was calculated as a ratio of the number of colonies to the number of cells plated (plating efficiency) divided by the same ratio calculated for the non-treated group.

### Radiation survival assay

HEp-2 cells were plated in 96-wells plates (2000 cells per well) and 60 mM dishes (1 × 10^5^ cells per dish). 10 nM triptolide was added until cells attached. After a 3h pre-treatment, cells were then radiated with various doses (0Gy, 2 Gy, 4 Gy, 6 Gy and 8 Gy) or 4 Gy alone at a dose rate of 300 cGy/min delivered by a Cs-137 Mark I irradiator. The control cells were treated with the same concentration of vehicle (0.01% DMSO) or mock IR. Cell viability assay and clonogenic assay were performed with the methods described above.

### Apoptosis assay

Apoptotic cells were analyzed as previously described [24]. HEp-2 cells grown on 6-well plates were treated with DMSO or various doses of triptolide for 24 h, and stained with Annexin V (AV) conjugated with FITC and propidium iodide (PI) using the Annexin V-FITC Apoptosis Assay Kit following the manufacturer’s instructions. Stained cells were analyzed with Cyflow Cube flow cytometer (PARTEC, Germany). Data were analyzed using FlowJO 7.6.5 software. 

### Real-time PCR

HEp-2 cells treated with various doses of triptolide for 24 h, then total RNA was isolated using RNAiso Plus Reagent according to the manufacturer’s instructions. 500 ng total RNA was reversely transcribed to cDNA using PrimeScript™ RT Master Mix (Perfect Real Time). Real-time PCR was performed on the Bio-Rad CFX 96 Real-time PCR system using SYBR^®^ Premix Ex Taq^TM^ II (Tli RnaseH Plus) and specific primers. The mRNA level of each gene was normalized to β-actin with ∆∆CT method using Bio-Rad CFX Manager V1.1.308.1111 software.

### Western Blot

Cell pellets were collected and lysed with RIPA lysis buffer containing 0.5 mM phenylmethylsulfonyl fluoride (PMSF) and protease inhibitors cocktail. The protein concentration of cell samples was analyzed by using BCA method. Equal amount proteins of each sample were electrophoresed on 8% to 15% SDS-PAGE gel and electrotransferred onto NC membrane. After incubation with appropriate primary and secondary antibodies, protein blots were detected by using ECL solution and ChemiDoc XRS+ imaging system (Bio-Rad, USA). β-actin or α-tubulin was used as loading control. 

### Immunoprecipitation (IP)

IP was carried out following the standard protocol provided by Cell Signaling Technology. Cell pellets were lysed as mentioned above by using NP-40 lysis buffer. Equal amount proteins of each sample were subjected to immunoprecipitation with anti-p53 primary antibody and Protein A/G plus-Agarose. Precipitated samples were electrophoresed on 10% or 15% SDS-PAGE gel and specific protein blots were detected with appropriate antibodies. 

### Immunofluorescence assay

HEp-2 cells were grown on sterilized glass coverslips overnight and treated with 50 nM Triptolide for desired times. After being fixed with 4% paraformaldehyde solution and blocked with 4% BSA in PBS, cells on coverslips were incubated with γ-H2AX primary antibody and anti-rabbit secondary antibody conjugated with Alex Flour 555, then stained with DAPI and transferred to a slide. Images were captured with a BX51+DP70 fluorescence microscope (Olympus, Japan).

### Statistical Analysis

Data were expressed as mean ± SD from three or more experiments. Statistical analysis was performed by using Student's t-test. Differences were considered statistically significant with p<0.05.
